# A Chromosome-Level Reference Genome of Chinese Balloon Flower *(Platycodon grandiflorus)*

**DOI:** 10.3389/fgene.2022.869784

**Published:** 2022-04-08

**Authors:** Yanyan Jia, Shaoying Chen, Weikai Chen, Ping Zhang, Zhenjing Su, Lei Zhang, Mengxin Xu, Li Guo

**Affiliations:** ^1^ School of Automation Science and Engineering, Faculty of Electronic and Information Engineering, Xi’an Jiaotong University, Xi’an, China; ^2^ School of Big Data, Weifang Institute of Technology, Weifang, China; ^3^ Peking University Institute of Advanced Agricultural Sciences, Weifang, China

**Keywords:** *platycodon grandiflorus*, genome assembly, Oxford nanopore, phylogenomics, Hi-C

## Introduction

Chinese balloon flower *(Platycodon grandiflorus)* is the sole species in genus Platycoldon within the Campanulaceae family. The typical blue purple or white flowers of *P. grandiflorus* are frequently used for ornamental purposes (Lv et al., 2021). As a traditional oriental medicine used to treat chronic inflammatory diseases, *P. grandiflorus* roots have rich pharmacological activities such as expectorant antitussive, anti-inflammatory, immune regulatory and anti-tumor effects (Choi et al., 2010; Nyakudya et al., 2014; Buchwald et al., 2020; Ke et al., 2020; Lee et al., 2020). The dried form of the Platycodi radix is officially listed as a traditional herbal medicine in the Chinese, Korean and Japanese Pharmacopoeia (Su et al., 2021). Platycodi radix is also being pickled in northeast China, and made into kimchi in the Korean Peninsula. The market demand of *P. grandiflorus* follows the development and application of medicine, food, health products, cosmetics, ornamental and other fields (Ji et al., 2020), and its market prospects are bright.

Over 100 secondary metabolites have been isolated from *P. grandiflorus* including triterpenoid saponins, flavonoids, polyphenols, polysaccharide and so on (Zhang et al., 2015; Qiu et al., 2019; Huang et al., 2021). So far, the pharmacological and metabolic pathways of the main active ingredient triterpenoid saponins have been studied (Kim et al., 2020; Kim et al., 2021; Yu et al., 2021). However, the molecular basis of biochemical pathways for *P. grandiflorus* secondary metabolites is overall poorly understood, hindering the progress of molecular breeding and metabolic engineering of *P. grandiflorus* towards increased production and utilization of its natural products. A high-quality genome assembly of the *P. grandiflorus* will significantly accelerate the genetic characterization of secondary metabolic pathways, their regulatory mechanisms and genome-assisted breeding.

Previously, a draft genome sequence of *P. grandiflorus* (2n = 2x = 18) was assembled using Illumina short reads by Kim et al. yielding a quite fragmented assembly with scaffold N50 of 277 kb (Kim et al. 2020). In this study, we assembled and annotated a chromosome-scale reference genome for *P. grandiflorus* cultivar XJD. This genome assembly has a total length of 622.86 Mb anchored to nine chromosomes with a high contiguity (contig N50 = 29.34Mb, scaffold N50 = 65.83 Mb), representing a significant improvement over the previously published draft genome of *P. grandiflorus* (Kim et al., 2020). The chromosome-scale genome assembly will advance our understanding of genome function and evolution of *P. grandiflorus*, and facilitate its molecular breeding and metabolic engineering.

## Results and Discussion

### Genome Assembly

To produce a chromosome-level genome assembly of *P. grandiflorus* cultivar XJD. We generated about 73 Gb Nanopore long reads with an average read length of 24 kb, 112 Gb Illumina paired-end short reads of 150 bp, and 311 Gb high-throughput chromatin conformation capture (Hi-C) sequencing data. The *P. grandiflorus* genome was estimated to be 642.38 Mb in length with a heterozygosity rate of 0.92% and a repeat content of 60% based on K-mer analysis of Illumina reads (Supplementary Table S1, Supplementary Figure S1). Nanopore long reads were first used to produce the draft assembly by NextDenovo, which was 622.86 Mb with a contig N50 of 29.34 Mb (Supplementary Table S2) after base correction by Pilon using Illumina reads. The quality of the genome assembly was evaluated by mapping Illumina short reads to the assembly with 99.3% of short reads mapped to 96.8% of the assembled genome. Furthermore, we performed BUSCO analysis, showing that the genome assembly captured 98.1% complete BUSCOs, including 95.5% single-copy and 2.6% duplicated (Supplementary Table S3) indicating that the genome assembly had high completeness.

**FIGURE 1:**
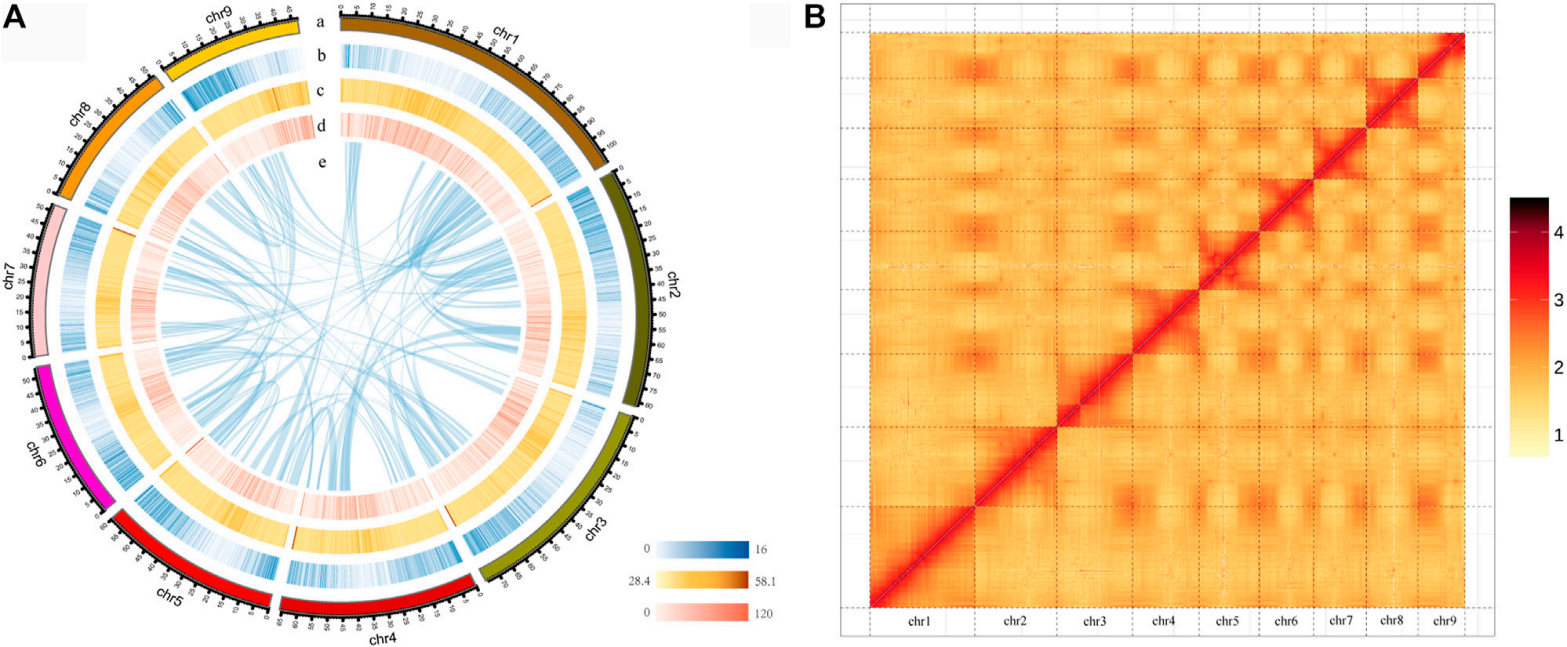
Overview of chromosome-level *Platycodon grandiflorus* genome assembly. **(A)** *P. grandiflorus* genomic features. Track a is the circular representation of nine pseudochromosomes. Track b-d represents the distribution of gene density, GC density, and repeat density, respectively, with densities calculated in 100 kb windows. Track e shows syntenic blocks identified within *P. grandiflorus* genome. **(B)** Hi-C interaction heatmap for the *P. grandiflorus* genome.

Hi-C data were then used to anchor the assembled contigs into individual chromosomes using ALLHiC (Zhang et al., 2019) and Juicerbox (Robinson et al., 2018), yielding nine pseudomolecules ranging from 47.09 to 104.37 Mb accounting for 95% of the assembly. Hi-C contact map showed that the nine pseudochromosomes could be distinguished clearly (Figure 1; Supplementary Table S4), consistent with the karyotype results (2n = 2x = 18) based on literature reports (Yang et al., 2016). The final genome assembly of *P. grandiflorus* was 622.86 Mb, with a contig N50 of 28.34 Mb, and a scaffold N50 of 65.83 Mb, the level of this genome assembly is much higher than a previous reported *P. grandiflorus* (Jangbaek-doraji cultivar) genome assembly (Kim et al., 2020) with a scaffold N50 of only 0.277 Mb (Supplementary Table S2). Whole genome sequence comparison showed that the two genome assemblies aligned well, where 4,815 scaffolds of Jangbaek-doraji assembly can be aligned to 99 scaffolds (95% anchored to nine chromosomes) of our XJD assembly (Supplementary Figure S2).

### Genome Annotation

We then performed genome annotations combining ab initio prediction, protein homology and transcriptome data from leaves, roots and stems (Methods). The genome annotation identified 360.46 Mb repeat sequences in the *P. grandiflorus* genome, accounting for 57.87% of the genome. The top two categories of repetitive elements were long terminal repeats (LTRs: 51.2%) and DNA elements (2.64%). A total of 22,358 protein-coding genes were predicted in the genome, 96.91% of which can be predicted gene function, by aligning against a library of known proteins in related plant species (Supplementary Table S5). Furthermore, non-coding RNAs were predicted across the *P. grandiflorus* genome, detecting a total of 1,867 microRNAs (miRNAs), 989 transfer RNAs (tRNAs), 780 ribosomal RNAs (rRNAs), and 1,114 small nuclear RNAs (snRNAs).

### Comparative Phylogenomics of *P. grandiflorus*

**FIGURE 2:**
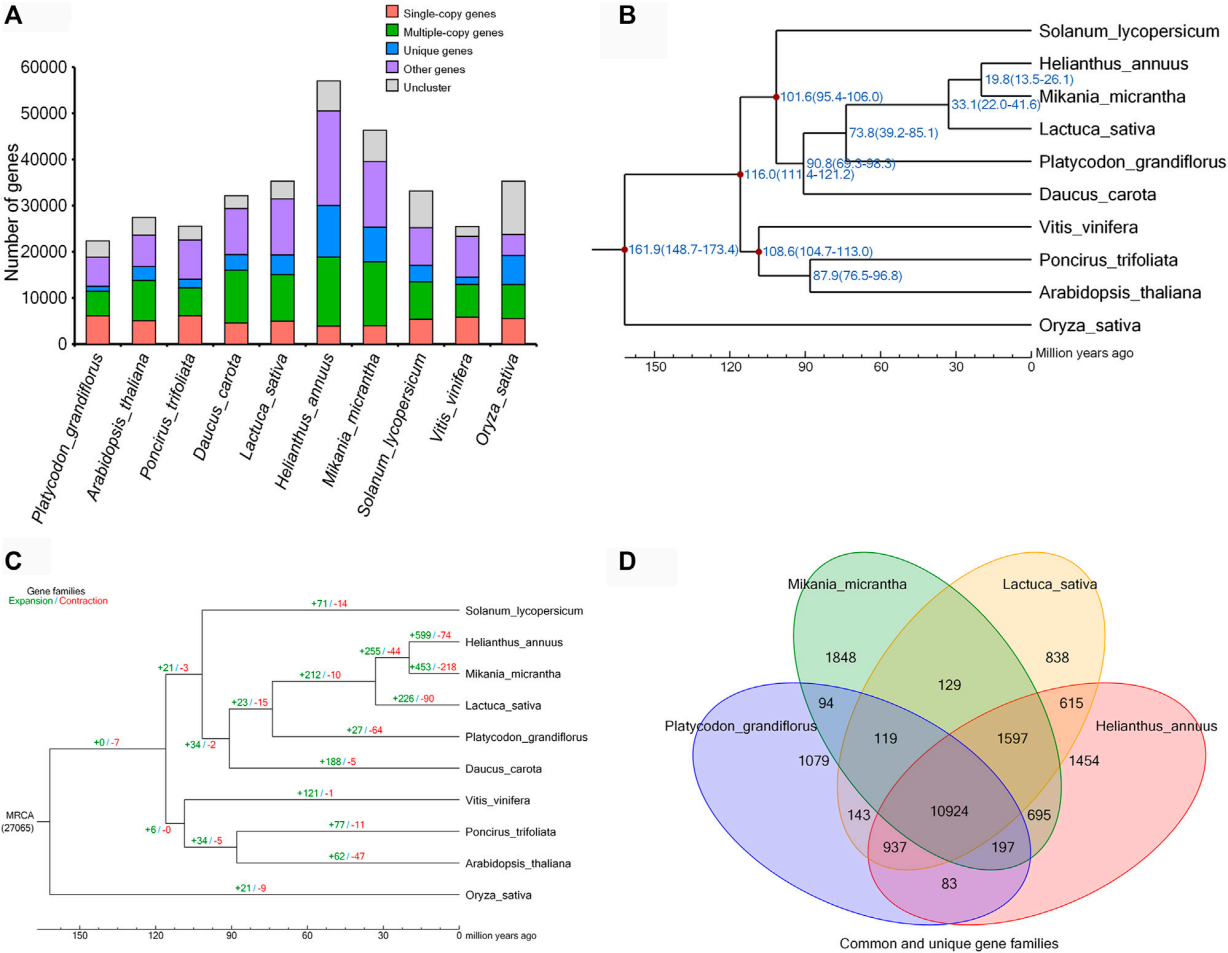
*Platycodon grandiflorus* phylogenomics. **(A)** The distribution of single-copy, multiple-copy, unique, and other genes in the 10 plant species. **(B)** Phylogenetic tree of the 10 plant species. The blue numbers denote divergence time of each node (MYA: million years ago). **(C)** Expansion and contraction in gene families of the 10 plant species. **(D)** Venn diagram represents the common and unique gene families among four closely related plants.

To determine the evolutionary relationships among *P. grandiflorus* and other species, we identified 1,436 single-copy orthologs from 10 representative plant species using OrthoMCL (Li et al., 2003) (Figure 2A). The protein sequence alignment of these orthologs were generated by MUSCLE (Edgar, 2004) and were used to generate a phylogenetic tree using Oryza sativa as outgroup (Figure 2B). *Mikania micrantha, Helianthus annuus, Lactuca sativa* were most closely related to *P. grandiflorus* with a divergence time around 73.8 million years ago (Mya) (Figure 2B). Gene family evolution analysis using CAFE on the 10 plant speices suggested that *P. grandiflorus* has 27 and 64 significantly expanded and contracted gene families (Figure 2C). Expansion gene families were enriched in 19 GO categories and 12 KEGG pathways, most of which were related to biosynthesis of secondary metabolites such as brassinosteroid, flavonoid, stilbenoid, and gingerol, and signaling pathway such as MAPK pathway (Supplementary Tables S6 and S7). Notably, *P. grandiflorus* contained 1,079 species-specific gene families consisting 1,914 genes relative to *M. micrantha, H. annuus and L. sativa* (Figure 2D). Then the GO enrichment analyses of these specific genes were performed (Supplementary Table S8). Positively selected genes in *P. grandiflorus* were identified by comparing with H. annuus and M. micrantha, the results of GO and KEGG analysis showed that the positively selected genes were significantly involved in DNA repair, cellular response to stress and stimulus, DNA metabolic process, nucleic acid metabolic process, DNA recombination, and so on (Supplementary Tables S9 and S10).

## Materials and Methods

### Plant Materials, Library Construction, and Sequencing

Fresh leaf, stem and root samples were collected from four-week-old seedlings of *P. grandiflorus* cultivar XJD grown in a plant growth chamber with a 16-h light photoperiod. The tissues were flash-frozen in liquid nitrogen and used for total genomic DNA or RNA extraction. Total genomic DNA of *P. grandiflorus* leaves were extracted using a DNeasy Plant Mini Kit (Qiagen), followed by PCR-free library construction using Illumina TruSeq DNA PCR-Free Library Preparation Kit following the manufacturer’s instructions. The libraries were sequenced on Illumina HiseqX Ten platform to generate 150 bp paired-end reads used to perform genome survey, polish the genome assembly, and evaluate the quality of assemblies.

For ONT and Hi-C sequencing, fresh young leaves were used for DNA isolation and library construction. For ONT sequencing, total genomic DNA was extracted from leaf samples using the CTAB method. ONT libraries were constructed and used for sequencing in the following steps: fragment repair, connecting reactions, quantitative detection, and library construction. Finally, single-molecule real-time sequencing was carried out on the Nanopore PromethION sequencer to obtain the raw data prior to error correction to obtain high fidelity sequence data. The Hi-C sequencing libraries were generated following a standard procedure described previously (Rao et al., 2014) involving crosslink DNA, restriction enzyme digestion, filling ends and biotin labeling, ligation, DNA purification and capture using antibody. The Hi-C libraries were subjected to quality control before being sequenced on Illumina HiseqX Ten platform. For transcriptome sequencing, total RNA was extracted from leaves, stems and roots of *P. grandiflorus* using the Plant RNA Purification Reagent (Qiagen) according to the manufacturer’s instructions. RNA-seq transcriptome libraries were prepared using the TruSeq RNA sample preparation Kit (Illumina), and sequencing was performed on an Illumina HiseqX Ten platform.

### De Novo Genome Assembly

K-mer frequency analysis was performed using Jellyfish V2.0 (Marçais and Kingsford, 2011) to estimate the *P. grandiflorus* genome size, heterozygosity and repeat content. The NextDenovo (https://github.com/Nextomics/NextDenovo) was used to assemble the *P. grandiflorus* genome with ONT long reads, and then the Nanopore-assembled genome was polished using the Illumina DNA short reads by NextPolish V1.3.1 (Hu et al., 2020) to improve base accuracy using default parameters. Next, the ALLHiC V0.9.8 (Zhang et al., 2019) was used to reorder and anchor preliminarily assembled contigs into chromosomes based on Hi-C data using default parameters. Finally, we use the Juicerbox V1.1 (Robinson et al., 2018) to adjust the heatmap and assemble it into a chromosome version of the genome. To assess the accuracy and completeness of the assemblies, Illumina clean reads were mapped to our assembly using BWA (Li and Durbin, 2009). In addition, BUSCO (Simão et al., 2015) was used to access the completeness of the genome assembly.

### Genome Annotation

Genome annotation mainly includes repetitive sequence annotation, gene annotation and non-coding RNA annotation. Firstly, transcriptome read assemblies were generated with Trinity (Grabherr et al., 2013) for the genome annotation. To optimize the genome annotation, the RNA-Seq reads from different tissues were aligned to draft genome using Hisat2 (Kim et al., 2015) with default parameters to identify exons region and splice positions. The alignment results were then used as input for Stringtie (Pertea et al., 2015) with default parameters for genome-based transcript assembly.

Repeat sequences were annotated based on homology and ab initio. Tandem Repeat was extracted using Tandem Repeats Finder (Benson, 1999) by ab initio prediction. RepeatModeler (Flynn et al., 2020), RepeatScout (Price et al., 2005), and LTR-Finder (Xu and Wang, 2007), were applied to ab initio repeat element library construction with default parameters, and RepeatMasker (Tarailo-Graovac and Chen, 2009) were used to annotate repetitive elements with the database. RepeatMasker and RepeatproteinMask were used to search the genome sequence for known repetitive elements, with the genome sequences used as queries against the repbase database (Jurka, 2000).

For gene structure prediction, Augustus (Stanke et al., 2008), GlimmerHMM (Majoros et al., 2004) and SNAP (Korf, 2004) were used in our *de novo* prediction study. Blast (Kent, 2002) and Genewise software (Birney et al., 2004) were used for homologous annotation performation. Based on homology prediction and *de novo* prediction results, combined with the transcriptome-based prediction data, the EvidenceModeler (Haas et al., 2008) was applied to integrate the prediction results for obtaining a non-redundant, more complete gene set. Finally, we used PASA (Haas et al., 2003), combined with the transcriptome assembly results, to correct the EVM annotation results, add UTR and variable shear and other information to get the final gene set. This final gene set was compared to public databases, including SwissProt (Bairoch and Apweiler, 2000), NR (Marchler-Bauer et al., 2011), Pfam (Griffiths-Jones et al., 2005), KEGG (Kanehisa et al., 2013), GO (Ashburner et al., 2000) and InterPro (Zdobnov and Apweiler, 2001) for function annotation of protein-coding genes. In addition, we also predicted different non-coding RNAs. The tRNAs were predicted using the program tRNAscan-SE (Chan and Lowe, 2019). For rRNAs are highly conserved, we predict rRNA sequences using BLAST. Other ncRNAs were identified by searching against the Rfam database with default parameters using the infernal software (Griffiths-Jones et al., 2005).

### Phylogenomic Analysis

Synteny analysis was conducted using MCScanX (Wang et al., 2012) applied to BLASTp results of *P. grandiflorus* protein sequences. For the phylogeny analysis, OrthoMCL (Li et al., 2003) was firstly used for detecting multi-copy gene families and single-copy gene families between *P. grandiflorus* and other representative species, and then all the single-copy gene families were performed for multiple sequence alignment using MUSCLE (Edgar, 2004), all the comparison results were combined together to form a super alignment matrix, RAxML (Stamatakis, 2014) was used to construct phylogenetic tree species. the Oryza sativa as an outgroup, and the bootstrap value was set to 100. The MCMCTREE of PAML (Yang, 1997) was implemented to estimate the differentiation time. Time correction points are: *Solanum lycopersicum - Helianthus annuus* (95–106 Mya), Vitis Vinifera - *Arabidopsis thaliana* (105–115 Mya), *P. grandiflorus - Vitis vinifera* (111–131 Mya), *P. grandiflorus–Oryza sativa* (148–173 Mya). The time correction points are taken from the TimeTree website (Sudhir et al., 2017).

### Gene Family Analysis

The CAFE software (Han et al., 2013) was used to analyze gene family expansion and contraction, based on the results of divergence times and phylogenetic relationships. In order to avoid false positive results, CAFE results were filtered, and the screening conditions for significant enrichment results were family-wide *p*-value < 0.05 and Viterbi *p*-value < 0.05. The enrichment analyses based on GO and KEGG annotations were performed to identify functional implications of the expanded and contracted genes.

### Positive Selection Analysis

The protein sequences of single-copy gene families were extracted and aligned by MUSCLE (Edgar, 2004). The Codeml program of PAML software was applied for positive selection analysis using the branch-site model with *H. annuus and M. micrantha* as the background branch. The likelihood ratio test was used to detect candidates that underwent positive selection with a cutoff *p* value of 0.05. Fisher’s test and FDR correction (q-value < 0.05) were used for functional enrichment analysis of these positively selected genes.

## Data Availability

The whole genome sequence data reported in this paper have been deposited in the Genome Warehouse in National Genomics Data Center (BioProject: PRJCA003843), Beijing Institute of Genomics, Chinese Academy of Sciences/China National Center for Bioinformation (GWH: GWHARYT00000000.1) publicly accessible at https://ngdc.cncb.ac.cn/gwh. The raw sequencing data for the ONT long reads, Illumina short reads, Hi-C Illumina and RNA-seq reads have been deposited in the Genome Sequence Archive at the National Genomics Data Center (GSA: CRA003503) publicly accessible at http://bigd.big.ac.cn/gsa. The genome annotation has been deposited in https://doi.org/10.6084/m9.figshare.19093331.v1.
