# Supplementary material for: A Chromosome-Level Reference Genome of Chinese Balloon Flower (Platycodon grandiflorus)
Source: Front Genet. 2022 Apr 8;13:869784. doi: 10.3389/fgene.2022.869784 (PMC9023762; doi:10.3389/fgene.2022.869784)
Supplement: Supplementary file 2 [file DataSheet1.DOCX]

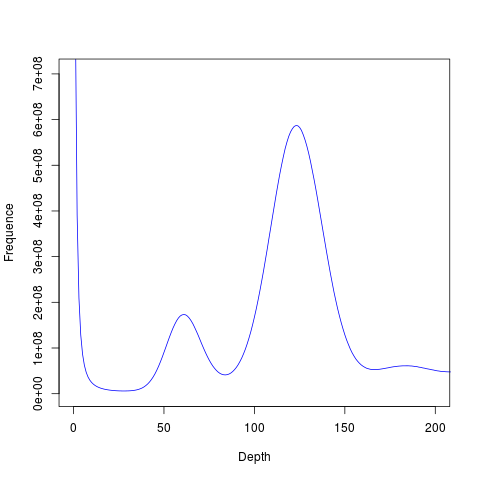


**Supplementary Figure 1.** Distribution of the number of the distinct K-mer (*K* = 17) of *Platycodon grandiflorus* XJD genome.


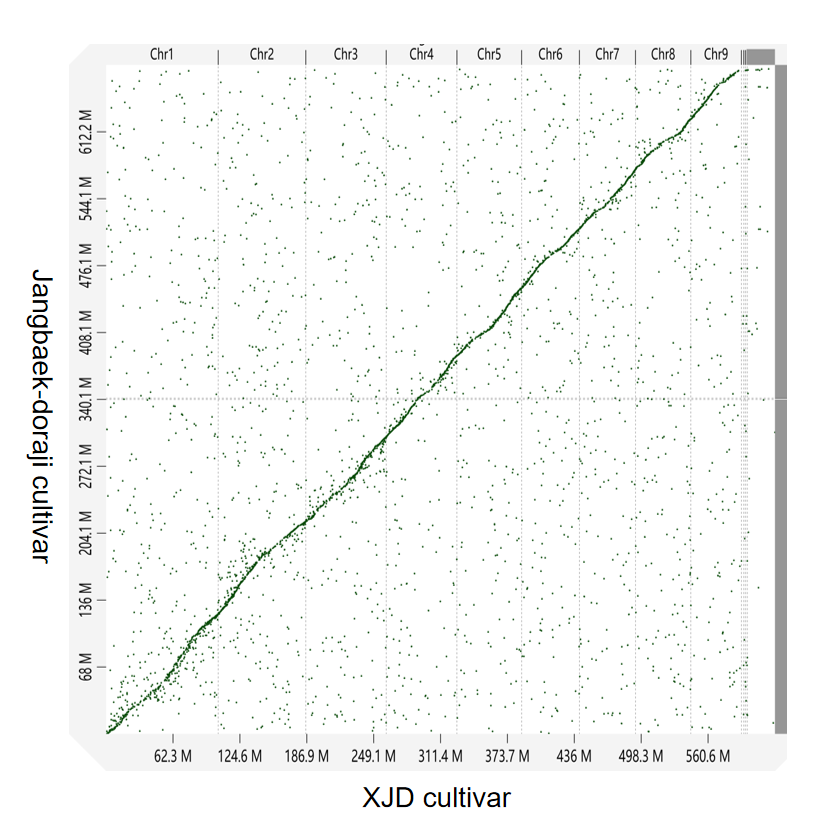


**Supplementary Figure 2.** The genome sequence dotplot of *P. grandiflorus* cultivar XJD and *P. grandiflorus* cultivar Jangbaek-doraji

**Supplementary Table 1.** Genomic characterization statistics based on 17-mer analysis.

| Kmer | Depth | N-kmer | Genomie size (Mb) | Heterozygous rate(%) | Repeat rate(%) |
| --- | --- | --- | --- | --- | --- |
| 17 | 122 | 79,206,291,711 | 649.23 | 0.92 | 60.00 |

**Supplementary Table 2.** Summary of the *Platycodon grandiflorus* genome assembly and annotation

| Assembly feature | XJD | Jangbaek-doraji ^a^ |
| --- | --- | --- |
| **Genome assembly** |  |  |
| Assembly size (Mb) | 622.86 | 680.1 |
| Pseudochromosomes | 9 | - |
| Total contigs | 167 | - |
| Contig N50 (Mb) | 28.33 | - |
| Total scaffolds | 99 | 4,815 |
| Scaffold N50 (Mb) | 65.83 | 0.277 |
| GC content (%) | 37.43 | 36.2 |
| BUSCO (%) | 98.1 | 96.9 |
| **Genome annotation** |  |  |
| Repetitive sequences (%) | 57.87 | - |
| Protein-coding genes | 22,358 | 40,017 |
| Annotated protein-coding genes | 21,667 | - |
| Non-coding RNAs | 4,750 | - |

^a^ A previous draft genome of *P. grandiflorus* var. Jangbaek-doraji (Kim et al., 2020).

**Supplementary Table 3**. The results of *P. grandiflorus* genomic quality assessment.

| BUSCO | Term | Number | Percentage(%) |
| --- | --- | --- | --- |
|  | Complete BUSCOs(C) | 1,583 | 98.1 |
|  | Complete and single-copy BUSCOs(S) | 1,541 | 95.5 |
|  | Complete Duplicated BUSCOs(D) | 44 | 2.6 |
|  | Fragmented BUSCOs(F) | 13 | 0.8 |
|  | Missing BUSCOs(M) | 18 | 1.1 |
| Short read coverage | Percentage of mapped reads | - | 99.28 |
|  | Coverage | - | 96.87 |

**Supplementary Table 4**. Statistics of cluster number and length of a single chromosome.

| Sequences ID | Cluster number | Sequences Length (bp) |
| --- | --- | --- |
| Hic_asm_0 | 15 | 104,366,066 |
| Hic_asm_1 | 7 | 81,560,240 |
| Hic_asm_2 | 6 | 74,919,121 |
| Hic_asm_3 | 3 | 65,833,826 |
| Hic_asm_4 | 14 | 60,262,457 |
| Hic_asm_5 | 6 | 53,822,365 |
| Hic_asm_6 | 3 | 52,243,604 |
| Hic_asm_7 | 14 | 51,522,710 |
| Hic_asm_8 | 9 | 47,088,837 |

**Supplementary Table 5**. Statistical results of gene function annotation in *P. grandiflorus*

| Term | Number | Percent (%) |
| --- | --- | --- |
| Total | 22,358 | - |
| Swiss prot | 18,028 | 80.63 |
| Nr | 21,315 | 95.34 |
| KEGG | 17,491 | 78.23 |
| InterPro | 21,375 | 95.60 |
| GO | 14,078 | 62.97 |
| Pfam | 17,579 | 78.63 |
| Annotated | 21,667 | 96.91 |
| Unannotated | 691 | 3.09 |

**Supplementary Table 6**. Functional enrichment of GO for significantly expanded gene families.

| GO_ID | GO_Term | GO_Class | Adjusted P-value | Count |
| --- | --- | --- | --- | --- |
| GO:0046914 | transition metal ion binding | MF | 1.54E-25 | 35 |
| GO:0008270 | zinc ion binding | MF | 6.43E-11 | 24 |
| GO:0043531 | ADP binding | MF | 1.68E-06 | 8 |
| GO:0007165 | signal transduction | BP | 1.26E-05 | 11 |
| GO:0005488 | binding | MF | 6.27E-05 | 81 |
| GO:0050794 | regulation of cellular process | BP | 6.52E-05 | 22 |
| GO:0050896 | response to stimulus | BP | 6.52E-05 | 16 |
| GO:0005506 | iron ion binding | MF | 6.52E-05 | 11 |
| GO:0016705 | oxidoreductase activity, acting on paired donors, with incorporation or reduction of molecular oxygen | MF | 0.000136542 | 11 |
| GO:0020037 | heme binding | MF | 0.00021118 | 11 |
| GO:0009733 | response to auxin | BP | 0.0003558 | 5 |
| GO:0043167 | ion binding | MF | 0.000850488 | 43 |
| GO:0003690 | double-stranded DNA binding | MF | 0.001699793 | 5 |
| GO:0055114 | oxidation-reduction process | BP | 0.002767208 | 19 |
| GO:0016491 | oxidoreductase activity | MF | 0.002767208 | 19 |
| GO:0016747 | transferase activity, transferring acyl groups other than amino-acyl groups | MF | 0.0032404 | 7 |
| GO:0006351 | transcription, DNA-templated | BP | 0.013342764 | 15 |
| GO:0003677 | DNA binding | MF | 0.019499123 | 15 |
| GO:0015935 | small ribosomal subunit | CC | 0.023504748 | 2 |

BP: Biological process; MF: Molecular function; CC: Cellular component.

**Supplementary Table 7**. Functional enrichment of KEGG for significantly expanded gene families.

| MapID | MapTitle | Adjusted P-value | Count |
| --- | --- | --- | --- |
| map00905 | Brassinosteroid biosynthesis | 1.13E-10 | 11 |
| map04010 | MAPK signaling pathway | 1.38E-10 | 25 |
| map00945 | Stilbenoid, diarylheptanoid and gingerol biosynthesis | 2.03E-06 | 7 |
| map00941 | Flavonoid biosynthesis | 0.00018373 | 7 |
| map04066 | HIF-1 signaling pathway | 0.003178626 | 5 |
| map03050 | Proteasome | 0.018221629 | 5 |
| map00710 | Carbon fixation in photosynthetic organisms | 0.026468057 | 5 |
| map00940 | Phenylpropanoid biosynthesis | 0.041776265 | 7 |
| map05010 | Alzheimer disease | 0.078364397 | 5 |
| map00010 | Glycolysis / Gluconeogenesis | 0.140361421 | 5 |
| map04940 | Type I diabetes mellitus | 0.153892976 | 2 |
| map00450 | Selenocompound metabolism | 0.246793085 | 2 |

**Supplementary Table 8**. Significantly enriched GO terms for specific family genes.

| GO_ID | GO_Term | GO_Class | Adjusted *P-value* |
| --- | --- | --- | --- |
| GO:0043531 | ADP binding | MF | 7.58E-18 |
| GO:0003690 | double-stranded DNA binding | MF | 2.50E-11 |
| GO:0000723 | telomere maintenance | BP | 1.89E-08 |
| GO:0008270 | zinc ion binding | MF | 4.83E-08 |
| GO:0042592 | homeostatic process | BP | 1.94E-06 |
| GO:0065007 | biological regulation | BP | 1.04E-05 |
| GO:0003678 | DNA helicase activity | MF | 1.22E-05 |
| GO:0004523 | RNA-DNA hybrid ribonuclease activity | MF | 1.27E-05 |
| GO:0046914 | transition metal ion binding | MF | 0.000123534 |
| GO:0005615 | extracellular space | CC | 0.000299201 |
| GO:2000112 | regulation of cellular macromolecule biosynthetic process | BP | 0.000458134 |
| GO:0019222 | regulation of metabolic process | BP | 0.000650749 |
| GO:0006355 | regulation of transcription, DNA-templated | BP | 0.000683379 |
| GO:0010468 | regulation of gene expression | BP | 0.000695185 |
| GO:0065008 | regulation of biological quality | BP | 0.00100977 |
| GO:0031323 | regulation of cellular metabolic process | BP | 0.001270406 |
| GO:0080090 | regulation of primary metabolic process | BP | 0.001432071 |
| GO:0050789 | regulation of biological process | BP | 0.001996047 |
| GO:0060255 | regulation of macromolecule metabolic process | BP | 0.002192182 |
| GO:0030915 | Smc5-Smc6 complex | CC | 0.0025457 |
| GO:0006357 | regulation of transcription | BP | 0.002638925 |
| GO:0006351 | transcription, DNA-templated | BP | 0.003258401 |
| GO:0033554 | cellular response to stress | BP | 0.003407487 |
| GO:0016717 | oxidoreductase activity | MF | 0.003457793 |
| GO:0050794 | regulation of cellular process | BP | 0.003550458 |
| GO:0030598 | rRNA N-glycosylase activity | MF | 0.003550458 |
| GO:0000724 | double-strand break repair | BP | 0.004854864 |
| GO:0004540 | ribonuclease activity | MF | 0.005238132 |
| GO:0006281 | DNA repair | BP | 0.005911515 |

BP: Biological process; MF: Molecular function; CC: Cellular component.

**Supplementary Table 9**. Functional enrichment of GO for significantly positive selection gene.

| GO_ID | GO_Term | GO_Class | Adjusted P-value | Count |
| --- | --- | --- | --- | --- |
| GO:0006281 | DNA repair | BP | 6.02E-05 | 13 |
| GO:0033554 | cellular response to stress | BP | 6.26E-05 | 15 |
| GO:0006259 | DNA metabolic process | BP | 9.78E-05 | 17 |
| GO:0090304 | nucleic acid metabolic process | BP | 0.000821175 | 42 |
| GO:0046483 | heterocycle metabolic process | BP | 0.000821175 | 47 |
| GO:0006950 | response to stress | BP | 0.000875636 | 16 |
| GO:0051716 | cellular response to stimulus | BP | 0.001196097 | 17 |
| GO:1901360 | organic cyclic compound metabolic process | BP | 0.001359842 | 47 |
| GO:0006139 | nucleobase-containing compound metabolic process | BP | 0.001359842 | 44 |
| GO:0034641 | cellular nitrogen compound metabolic process | BP | 0.001456145 | 46 |
| GO:0006310 | DNA recombination | BP | 0.001456145 | 6 |
| GO:0006725 | cellular aromatic compound metabolic process | BP | 0.001456145 | 46 |
| GO:0006396 | RNA processing | BP | 0.001713976 | 15 |
| GO:0006807 | nitrogen compound metabolic process | BP | 0.002876355 | 48 |
| GO:0003887 | DNA-directed DNA polymerase activity | MF | 0.027423192 | 4 |
| GO:0006298 | mismatch repair | BP | 0.027423192 | 3 |
| GO:0030983 | mismatched DNA binding | MF | 0.029946019 | 3 |
| GO:0003676 | nucleic acid binding | MF | 0.030535774 | 41 |

BP: Biological process; MF: Molecular function

**Supplementary Table 10**. Functional enrichment of KEGG for significantly positive selection gene.

| MapID | MapTitle | Adjusted P-value | Count |
| --- | --- | --- | --- |
| map03460 | Fanconi anemia pathway | 0.018595409 | 7 |
| map04214 | Apoptosis - fly | 0.247193088 | 4 |
| map03430 | Mismatch repair | 0.281258485 | 4 |
| map03440 | Homologous recombination | 0.281258485 | 5 |
| map02026 | Biofilm formation - Escherichia coli | 0.281258485 | 2 |
| map03040 | Spliceosome | 0.281258485 | 9 |
| map05224 | Breast cancer | 0.281258485 | 2 |
| map05212 | Pancreatic cancer | 0.281258485 | 2 |
| map04668 | TNF signaling pathway | 0.281258485 | 3 |
| map05203 | Viral carcinogenesis | 0.281258485 | 6 |
| map00053 | Ascorbate and aldarate metabolism | 0.285054712 | 3 |
